# Post-Mortem Corneal Thickness Measurements with a Portable Optical Coherence Tomography System: a Reliability Study

**DOI:** 10.1038/srep30428

**Published:** 2016-07-26

**Authors:** Pietro Emanuele Napoli, Matteo Nioi, Ernesto d’Aloja, Maurizio Fossarello

**Affiliations:** 1 Department of Surgical Sciences, Eye Clinic, University of Cagliari, Cagliari, Italy; 2 Department of Public Health, Clinical and Molecular Medicine–Forensic Science Unit–University of Cagliari, Cagliari, Italy

## Abstract

The purpose of this study was to determine the repeatability and reproducibility of post-mortem central corneal thickness (CCT) measurements by using a real-time, portable optical coherence tomography (OCT) system on an animal model, and to prospectively evaluate the time-course of post-mortem changes in CCT. Forty-six ocular globes of sheep (*Ovis aries*) were analyzed with a portable spectral-domain OCT device by two operators at different postmortem intervals (PMIs) as follows: *immediately* (i.e. within 10 minutes), at the 30^th^ minute, at the 1^st^, 6^th^, 12^th^, 24^th^ and 48^th^ hour, and *later* (up to the 96^th^ hour). The coefficient of repeatability ranged from 0.3% to 3.5%, and coefficients of reproducibility ranged from 0.2% to 3.7% in the central region of the cornea. The intraclass correlation coefficients were particularly high at different PMIs, thus confirming good measurement reliability with the portable OCT. The average CCT decreased immediately and then increased thereafter, with two peaks at 6 and 24 hours after death. Our results suggest that portable OCT is a reliable tool for monitoring CCT variations after death and may be useful in characterizing corneas before explantation, detecting quantitative variations during post-mortem corneal degeneration or assessing changes in CCT for forensic implications.

Over the past two decades, optical coherence tomography (OCT) has revolutionized basic research on various biological systems^1^,^2^. In particular, at present it represents a fast, non-invasive, high-resolution, contactless, light-based imaging modality for exploring cross-sectional, subsurface areas of several ocular microstructures in real time^3^,^4^,^5^,^6^,^7^. Although the basic assumption in achieving reliable OCT measurements on ocular tissues is the *visual fixation* (VF) of the patient at a fixation target, in some cases this condition is impossible to obtain (e.g. for blind or visually-impaired patients, or for post-mortem studies of ocular changes)^8^.

*In vivo* OCT quantitative evaluation of central corneal thickness (CCT) has different important applications for management of corneal diseases, glaucoma, and refractive surgery^9^,^10^. Then again, OCT central pachymetry measurements during various post-mortem intervals (PMIs) may have a potential for obtaining ultrastructural information for the selection of corneas for transplantation, for gaining a more comprehensive knowledge of pathophysiological processes of corneal degeneration at each layer, as well as for a possible estimation of time since death for forensic sciences^11^,^12^,^13^.

However, to our knowledge, no study has been published on the intra- and inter-observer reliability of post-mortem CCT measurements taken *in situ* with OCT. To evaluate changes in corneal thickness after death it is clearly necessary first to use a portable OCT system (which can be used with patients in the *supine position*) at different PMIs (i.e. without VF) to quantify the level of reproducibility and repeatability of CCT measurements, as well as to evaluate the temporal range in which this quantitative approach can be reliably applied. The purpose of this study was to determine the repeatability and reproducibility of post-mortem CCT measurements by using a real-time, portable OCT system on an animal model. In addition, we evaluated the time-course of post-mortem changes in corneal thickness.

## Methods

### Collection of samples

Sheep heads of young adult animals (*Ovis aries*) that passed the standard controls for food consumption were obtained after animal sacrifice from a local slaughterhouse (CO.AL.BE. dei F.lli Contu & C. S.n.c. Selargius, Cagliari, Sardinia, Italy). Sheep heads normally represent waste material, so there was no need for an *ad hoc* animal protocol nor were there associated costs. Furthermore, the choice of sheep samples was driven by the fact that we knew, in real time, the *exact* moment of death in order to perform OCT scans for early and late (>48 h since death), *well-known* post-mortem intervals. Indeed, it may be difficult to exactly estimate the time since death in uncontrolled cases, as well as being forbidden by the legislation of different countries to study more than 48 h human cadavers (e.g., in this sense the Italian legislation is based on the *Regolamento di polizia mortuaria, DPR 285/90*).

In total, forty-six ocular globes were analyzed with a portable spectral-domain OCT (SD-OCT) system (iVue SD-OCT, Optovue Inc, Fremont, CA). All examinations were conducted in the same conditions of temperature (within a range of 12–22 °C) and humidity (within a range of 50–60%) in a dimly lit room.

### Optical coherence tomography (portable OCT system)

The iVue SD-OCT uses a superluminescent diode scan with a center wavelength of 840 ± 10 nm to provide high-resolution images. This commercially available OCT system works at an image acquisition rate of 25,000 axial scans per second, a frame rate of 256 to 1024 A-scan/frame, and has a 5-μm axial resolution. The cross-sectional corneal images were acquired using the pachimetry mapping protocol. The mode acquires a set of eight radial lines of equal length at 6 mm (22.5° interval). Each line is composed of 1020 A-scans. All SD-OCT scans were obtained through undilated pupils. Two experienced examiners (P.E.N, M.N.) scanned all ocular globes. All measurements were defined automatically by the iVue software. For our purposes, the reliability analysis focused on *central* corneal thickness (0–2 mm).

### Experimental procedure and OCT measurements

**Figure 1:**
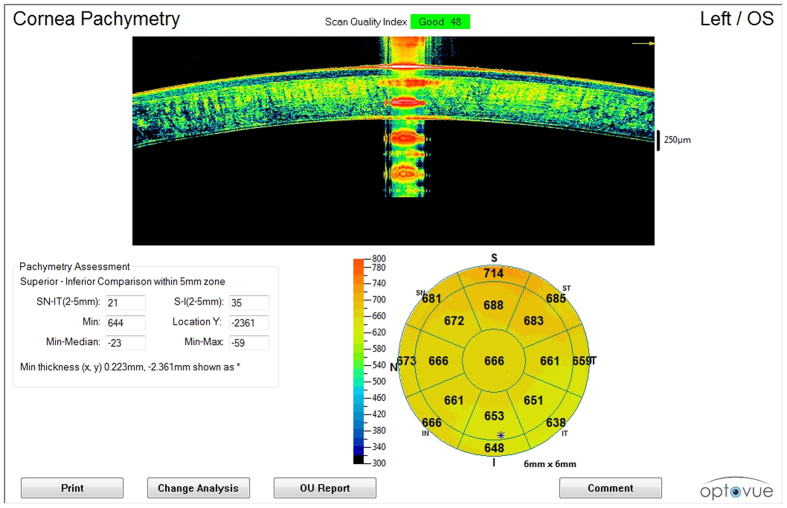
OCT imaging (pachymetry map). The iVue SD-OCT pachymetric map generated by each scan performed during various post-mortem intervals. Central corneal *reflex* and alignment beam were coaxial during OCT scanning (at the top of the image). *Central pachymetry* was obtained in the central region, 0–2 mm in diameter (at the bottom right of the image).

During each measurement session, the sheep’s head position (maintained by a mechanical support) and corneal reflex were monitored and adjusted so that the central corneal reflex and alignment beam were coaxial (Fig. 1). The eyes were kept open during OCT imaging (lids were temporarily sutured with adhesive bandage strips).

The OCT scans were performed at different time-points since death (also defined as sessions) as follows: *immediately* (or *baseline*, i.e. within 10 minutes), at the 30^th^ minute, at the 1^st^, 6^th^, 12^th^, 24^th^ and 48^th^ hour, and *later* (up to the 96^th^ hour, unless images were uninterpretable).

Ocular globes underwent scanning sessions with a pachimetry mapping protocol as follows: two scans were performed by operator 1 (MN) and one further scan by operator 2 (PEN) for each time-point to determine intra- and inter-observer reproducibility. Each measurement session for each time-point was completed within 2–3 minutes, otherwise it was excluded. For paired intraobserver measurements, no more than 1 minute elapsed between the first and second measurements. For paired interobserver measurements, no more than 2 minutes elapsed between the first and second measurement.

Rough alignment was achieved by centering the cornea on the real-time video image, and fine alignment was adjusted using a horizontally directed scan. Cross-sectional scans were displayed continuously on the PC monitor. The operator adjusted the system to position the vertex at the center of the OCT image and maximize the vertex reflection. Acceptable scans were selected as soon as they appeared. Images were judged to be of *adequate quality* based on the following criteria: good demarcation of the anterior and posterior corneal boundaries and absence of artifacts owning to eyelid margin. Moreover, quality assurance checks were performed and images that were poorly focused, were not centered, or had a *scan quality index* of less than 30 were excluded. Sheep heads were repositioned between OCT scans.

## Statistical Analysis

Mean and SD of average CCT measurements (0–2 mm) in each of the post-mortem time-points were computed for each observer. Coefficients of repeatability and reproducibility, as well as intraclass correlation coefficients (ICCs), were determined for the reliability analysis. As proposed by the British Standard Institution and recommended by Bland and Altman, the repeatability coefficient was defined as 2 SDs of the differences between pairs of measurements on the same ocular globe obtained at the same time-point by the same observer divided by the average of the means of each pair of readings^14^,^15^,^16^. Similarly, the reproducibility coefficient was defined as 2 SDs of the difference between measurements obtained during repetition of the test in the same ocular globe and for the same time-point by different observers. A graph of differences against means was plotted for the overall CCT averaged over the intraobserver/interobserver results.

Coefficients of variation for intraobserver and interobserver measurements were obtained by taking the within-subject SD divided by the within-subject mean. ICC was calculated on the basis of the analysis of variance for mixed models for each situation as proposed by Bartko and Carpenter^17^.

Values close to 1 indicated high repeatability/reproducibility of the post-mortem CCT measurements.

The Wilcoxon matched-pairs test (5% significance level) was also used to determine whether there was any statistically significant difference (intraobserver and/or interobserver) between measurements obtained for each PMI session.

Statistical significance was defined as *P* < 0.05. Statistical analyses were performed with a commercially available statistical software package (SPSS for Windows, version 21.0; SPSS Inc, Chicago, IL).

## Results

The mean and SDs of post-mortem CCT measurements in the central region (0–2 mm) obtained for each PMI and for the 46 eyes are shown in Table 1 (in micrometers). Overall, a total of 1104 post-mortem CCT measurements were performed. The coefficients of variation and repeatability/reproducibility for each PMI are shown in Table 2. The ICCs for each PMI as estimates of intraobserver/interobserver reproducibility are also shown in Table 2.

**Figure 2:**
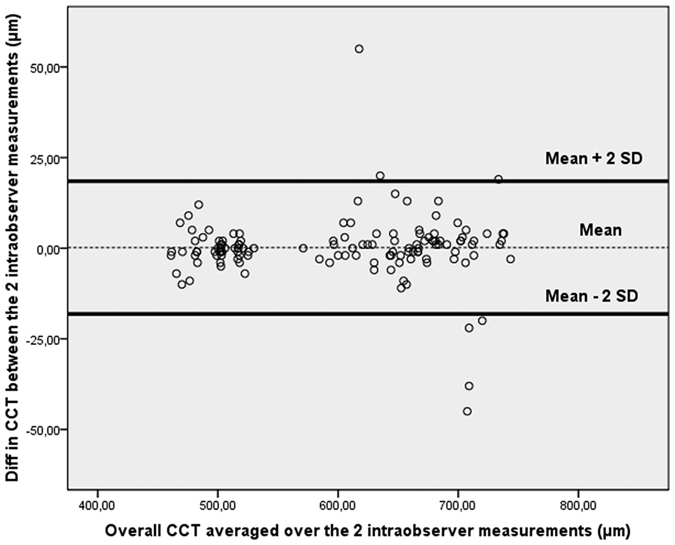
Bland-Altman plot of repeatability of central corneal thickness (CCT) measurements. Mean central corneal thickness (CCT) for paired intraobserver measurements (obtained by operator 1) is plotted against the difference in CCT between the two results. Overall, 95% of the values fell within 2 SDs of the mean.

**Figure 3:**
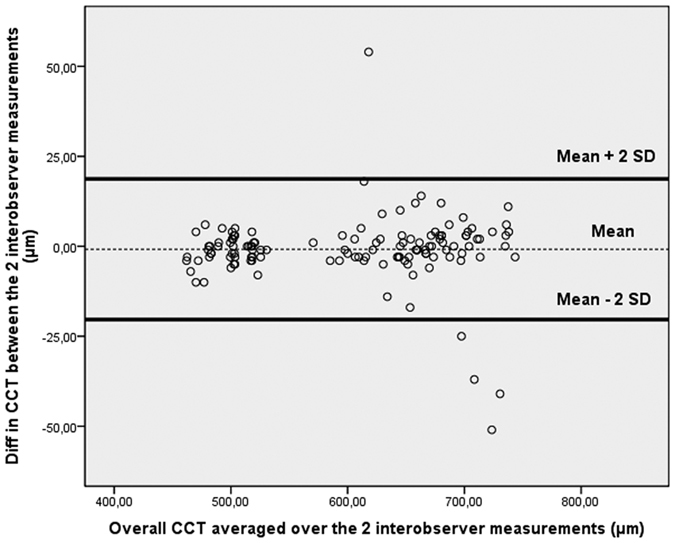
Bland-Altman plot of reproducibility of central corneal thickness (CCT) measurements obtained by observer 1 (first measurement) and observer 2. Mean central corneal thickness (CCT) for paired interobserver measurements (obtained by operator 1 and operator 2) is plotted against difference in CCT between the two results. Ninety-five percent of the values fell within 2 SDs of the mean.

**Figure 4:**
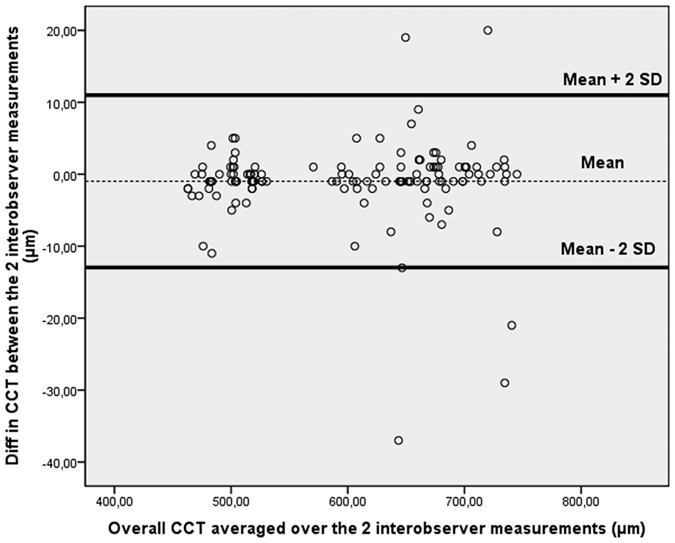
Bland-Altman plot of reproducibility of central corneal thickness (CCT) measurements obtained by observer 1 (second measurement) and observer 2. Mean central corneal thickness (CCT) for paired interobserver measurements (obtained by operator 1 and operator 2) is plotted against difference in CCT between the two results. Ninety-six percent of the values fell within 2 SDs of the mean.

Graphs of differences against means were plotted for the CCT measurements obtained by operator 1 and/or 2 (Figs 2, 3, 4). In all cases it was found that ≥95% of differences fell within 2 SDs of the mean. This result indicates reproducibility in post-mortem CCT measurements according to the definitions of the British Standards Institution. There was no significant association between the within-subject SD and mean intraobserver, interobserver, or intrasession corneal thickness measurements. The Wilcoxon matched-pairs test showed no significant systematic differences between measurements obtained for each PMI by observers. There were no missing or excluded data.

**Figure 5:**
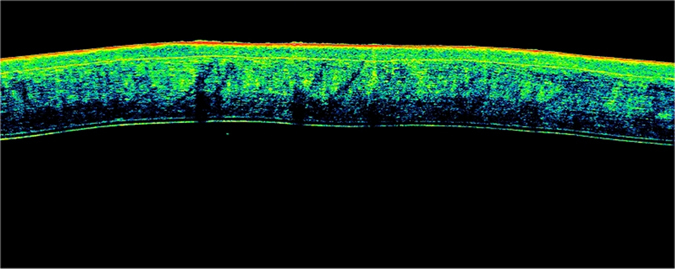
OCT imaging of corneal morphology in later post-mortem intervals. A horizontal, high-resolution Fourier-domain, cross-sectional OCT scan of the central cornea (2 mm of diameter). Irregular variations in *corneal contour* due to corneal edema, ocular hypotony or advanced decomposing processes were observed during OCT scans performed after 48 hours from death. These findings probably led to an increase in variability of pachymetric measurements obtained during later post-mortem intervals (within a range of 48–96 hours) compared with earlier results.

**Figure 6:**
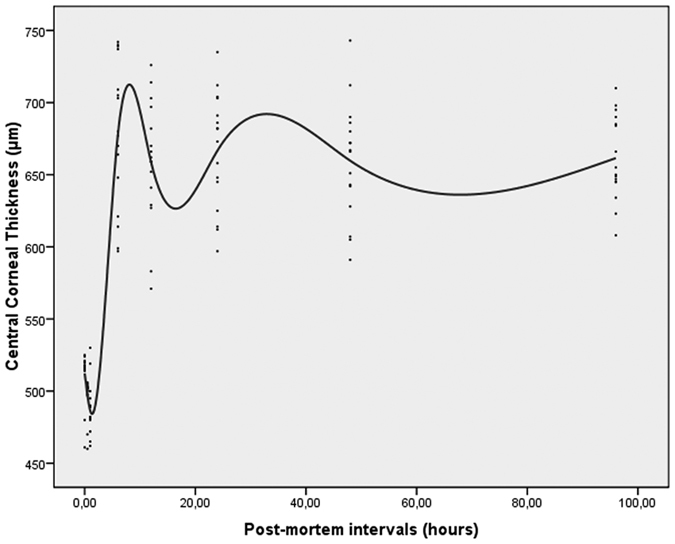
Central corneal thickness (CCT) determined by optical coherence tomography versus post-mortem intervals. Corneal thickness decreases immediately after death and increases thereafter, with two different peaks at 6 and 24 hours after death.

The corneal shape and thickness variations are reported in Figs 1, 5 and 6. As can be seen, the corneal thickness decreases immediately after death, only to increase thereafter, with two different peaks at 6 and 24 hours after death (Fig. 6).

## Discussion

In the present study, the portable iVue SD-OCT system provided fast, high-resolution quantitative and qualitative imaging of the cornea during various PMIs. This imaging technique may be useful in monitoring corneal changes after death, for a potential microstructural analysis during donor selection for cornea transplantation, and for a quantitative evaluation of human forensic post-mortem intervals. However, the feasibility of this instrument in exploring CCT changes during various PMIs strongly depends on the reliability of its measurements. The aim of this work was therefore to determine the repeatability and reproducibility of CCT measurements with the pachymetry mapping protocol of a commercially available portable OCT system.

The data from our study demonstrate that post-mortem CCT measurements obtained with the pachymetry mapping protocol of iVue SD-OCT are repeatable and reproducible. Measurements performed on an animal model showed that the repeatability coefficient ranged from 0.3% to 3.5%, and the reproducibility coefficient ranged from 0.2% to 3.7% in the central region of the cornea. The ICCs were also significantly high during different PMIs, thus demonstrating good reliability of measurements with iVue SD-OCT. Moreover, intrasession coefficients of variation of less than 1.9% showed the high level of intrasession reproducibility.

Reliability of post-mortem CCT measurements are dependent on such factors as intra/inter-operator precision in positioning over the central cornea during scanning, irregularity in corneal thickness along neighboring points and amount of sampling points for each area.

It is interesting to note that measurements in the central cornea during later PMIs (>48h) showed less reliability compared with earlier measurements. In these cases, slight variations in scan positioning resulted in an increased variability of CCT measurements. These findings are probably related to variations in the corneal morphology observed (Fig. 5), such as the corneal contour due to corneal edema, ocular hypotony or advanced decomposing processes.

Nevertheless, our data demonstrate that repeatability and reproducibility remained excellent until the 48^th^ hour after death, with coefficients of variation/repeatability/reproducibility of less than 1.2%. Measurement reliability in post-mortem changes occurring at a later stage might be expected to increase if corneal degeneration/putrefaction processes are halted by favorable environmental conditions (e.g. lower storage temperatures).

Of note, the operator’s consistency in positioning over the central cornea during different scans is facilitated by means of iVue SD-OCT, since it allows continuous monitoring of the ocular surface during scanning and of the corneal reflex from the vertex for a proper centration.

The reliability of post-mortem CCT measurements with iVue SD-OCT is also facilitated by *automated scan processing* that allows correction for image distortion due to refractive index transition at the air–cornea interface, as well as by a *computer algorithm* that identifies signal peaks of anterior and posterior corneal boundaries. With regard to the acquisition time of OCT scans, the pachymetry mapping protocol, consisting of eight *radial lines* centered on the cornea, requires approximately 0.5 seconds. This clearly implies a slightly longer acquisition time than a *single line scan* (0.125 seconds), but it is still rapid.

Although *spot pachymetry* (ultrasound pachymetry) used by previous authors for post-mortem measurements is easy to perform and inexpensive, it offers only spot measurements, highly dependent on the operator’s probe placement^11^. Conversely, iVue-OCT provides high resolution, cross-sectional tomographic imaging of a 3.14-mm^2^ *area* of the central cornea (2 mm in diameter), by measuring backscattered light. Moreover, it permits a precise definition of anterior and posterior corneal boundaries and a morphological study of the corneal tissue.

The present study has some limitations. Although our data prove the reliability of portable OCT for *in situ* pachymetric measurements, future studies should verify our quantitative and qualitative approach in humans. Moreover, it would be interesting to compare post-mortem pachymetric measurements obtained by portable OCT versus different kinds of ultrasonic pachymeters, which are potentially cheaper and more accessible to eye bank services.

In conclusion, portable OCT offers forensic physicians and ophthalmologists a fast and manageable method for determining CCT in different PMIs with a significant level of reproducibility and repeatability. These results are particularly useful, since the increase in variability during late results is likely to be caused by actual changes in CCT rather than by measurement errors of the portable OCT system. The data of our study also indicate that portable OCT represents a reproducible method for monitoring early intervals after death, which may be useful for characterizing the corneas before explantation, detecting quantitative variations during post-mortem corneal degeneration or assessing changes in CCT for forensic implications.

## Additional Information

**How to cite this article**: Napoli, P. E. *et al*. Post-Mortem Corneal Thickness Measurements with a Portable Optical Coherence Tomography System: a Reliability Study. *Sci. Rep.* **6**, 30428; doi: 10.1038/srep30428 (2016).

## Figures and Tables

**Table 1:** Average Corneal Thickness Measurements.

PMIs (timepoints)	M1	M2	M3	Δ (M1–M2)	Δ (M1–M3)	Δ (M2–M3)
*Immediately*	512.31 ± 16.31	512.56 ± 16.68	513.50 ± 16.34	0.16 ± 9.15	−0.82 ± 9.77	−0.98 ± 5.98
*30*^*th*^ *minute*	497.19 ± 12.9	419.81 ± 12.5	419.75 ± 11.53	−0.62 ± 1.74	−0.56 ± 3.48	−0.06 ± 2.86
*1*^*st*^ *hour*	486.38 ± 18.04	486.37 ± 18.6	488.25 ± 18.19	0.0 ± 6.58	−1.87 ± 4.96	−1.87 ± 3.73
*6*^*th*^ *hour*	677.81 ± 50.83	675.19 ± 50.38	675.25 ± 49.85	2.62 ± 3.64	2.56 ± 3.79	−0.06 ± 1.48
*12*^*th*^ *hour*	658.81 ± 42.75	659.63 ± 41.19	659.19 ± 41.06	−0.81 ± 3.97	−0.37 ± 5.89	0.43 ± 3.4
*24*^*th*^ *hour*	666.69 ± 40.13	664.75 ± 40.24	664.69 ± 39.14	1.93 ± 3.33	2.0 ± 3.82	0.06 ± 2.01
*48*^*th*^ *hour*	659.69 ± 40.21	658.88 ± 36.73	660.75 ± 38.02	0.81 ± 6.4	−1.06 ± 4.4	−1.87 ± 3.0
Later*	661.5 ± 29.3	663.87 ± 47.09	667.56 ± 49.33	−2.37 ± 23.63	−6.06 ± 25.13	−3.68 ± 15.43

Data are expressed at the mean ± SD (in micrometers).

M1 represents measurements by observer 1.

M2 represents repeat measurements by observer 1 at the same session (time-point).

M3 represents repeat measurements by observer 2 at the same session (time-point).

Δ represents difference.

PMIs represent postmortem intervals since death.

*Within a range of 48–96 hours.

**Table 2:** Intraclass Correlations and Coefficients of Repeatability and Reproducibility.

PMIs (time-points)	ICC^a^ (M1–M2)	ICC^a^ (M1–M3)	ICC^a^ (M2–M3)	CoR % (M1–M2)	CV_W_%	CoR % (M1–M3)	CV_W_%	CoR % (M2–M3)	CV_W_%
*Immediately*	0.991 (0.974–0.997)	0.991 (0.975–0.997)	0.997 (0.993–0.999)	1.1 (0.8–1.3)	0.5 (0.4–0.7)	1.1 (0.8–1.3)	0.5 (0.4–0.7)	0.6 (0.4–0.8)	0.3 (0.2–0.4)
*30*^*th*^ *minute*	0.991 (0.973–0.997)	0.96 (0.888–0.986)	0.972 (0.921–0.99)	0.3 (0.2–0.5)	0.2 (0.1–0.3)	0.8 (0.6–1.1)	0.4 (0.3–0.5)	0.6 (0.3–0.9)	0.3 (0.2–0.4)
*1*^*st*^ *hour*	0.935 (0.826–0.977)	0.962 (0.896–0.987)	0.979 (0.942–0.993)	1.5 (1.0–2.1)	0.8 (0.5–1.0)	1.1 (0.6–1.6)	0.5 (0.3–0.8)	0.7 (0.2–1.0)	0.4 (0.1–0.6)
*6*^*th*^ *hour*	0.997 (0.993–0.999)	0.997 (0.992–0.999)	1.0 (0.999–1.0)	0.7 (0.4–1)	0.4 (0.2–0.5)	0.7 (0.4–1.0)	0.4 (0.2–0.5)	0.2 (0.1–0.3)	0.1 (0.0–0.2)
*12*^*th*^ *hour*	0.996 (0.987–0.998)	0.990 (0.972–0.997)	0.997 (0.99–0.999)	0.6 (0.3–0.9)	0.3 (0.1–0.4)	0.8 (0.3–1.3)	0.4 (0.1–0.6)	0.4 (0.1–0.7)	0.2 (0.1–0.4)
*24*^*th*^ *hour*	0.997 (0.99–0.999)	0.995 (0.987–0.998)	0.999 (0.996–1.0)	0.5 (0.2–0.9)	0.2 (0.1–0.4)	0.6 (0.2–0.9)	0.3 (0.1–0.4)	0.3 (0.1–0.5)	0.1 (0.1–0.2)
*48*^*th*^ *hour*	0.986 (0.961–0.995)	0.994 (0.982–0.998)	0.997 (0.991–0.999)	0.8 (0.3–1.3)	0.4 (0.1–0.6)	0.8 (0.5–1.0)	0.3 (0.2–0.5)	0.5 (0.2–0.7)	0.2 (0.1–0.4)
Later*	0.819 (0.556–0.933)	0.808 (0.534–0.928)	0.949 (0.860–0.982)	3.5 (1.7–5.4)	1.7 (0.8–2.7)	3.7 (1.6–5.7)	1.8 (0.8–2.8)	2.2 (1–3.5)	1.1 (0.5–1.7)

^a^Type C intraclass correlation coefficients using a consistency definition: the between- measure variance is excluded from the denominator variance. The estimator is the same, whether the interaction effect is present or not.

^*^Within a range of 48–96 hours.

Data in parentheses represent 95% CI. M1 represents measurements by observer 1; M2 represents repeat measurements by observer 1 at the same session (time-point); M3 represents repeat measurements by observer 2 at the same session (time-point). CoR (M1, M2) coefficient of repeatability; CoR (M1, M3), CoR (M2, M3), coefficients of reproducibility; CVw, coefficient of variation. CoR and CVw are expressed as percentages (Coefficients % = Coefficients *100).
